# On consensus biomarker selection

**DOI:** 10.1186/1471-2105-8-S5-S5

**Published:** 2007-05-24

**Authors:** Janusz Dutkowski, Anna Gambin

**Affiliations:** 1 Institute of Informatics, Warsaw University. Banacha 2 02-097 Warsaw, Poland

## Abstract

**Background:**

Recent development of mass spectrometry technology enabled the analysis of complex peptide mixtures. A lot of effort is currently devoted to the identification of biomarkers in human body fluids like serum or plasma, based on which new diagnostic tests for different diseases could be constructed. Various biomarker selection procedures have been exploited in recent studies. It has been noted that they often lead to different biomarker lists and as a consequence, the patient classification may also vary.

**Results:**

Here we propose a new approach to the biomarker selection problem: to apply several competing feature ranking procedures and compute a consensus list of features based on their outcomes. We validate our methods on two proteomic datasets for the diagnosis of ovarian and prostate cancer.

**Conclusion:**

The proposed methodology can improve the classification results and at the same time provide a unified biomarker list for further biological examinations and interpretation.

## Background

There is great hope among clinical proteomics researchers that mass spectrometry (MS) will soon become a powerful diagnostic tool. Extensive research has been conducted on statistical learning methods for disease prediction from MS data (see e.g. [1-9]). The data considered in this context are mainly spectra of complex peptide mixtures, such as plasma or serum samples. These spectra contain signals of thousands of peptides. The dimension of the input space is therefore very high, while the number of available samples is relatively small (a few hundred at most). Furthermore, only a small fraction of the peptides may potentially be significant in determining the health state of the patient. Most classification algorithms do not perform well in this setting, and for the ones that do (e.g. random forests) the results become difficult to interpret. Here we consider two paradigms for reducing the dimension of the data and identifying informative variables: feature selection and feature extraction. We review commonly used feature selection and extraction procedures and propose a new approach based on aggregating the preferences of several competing methods.

**Figure 1:**
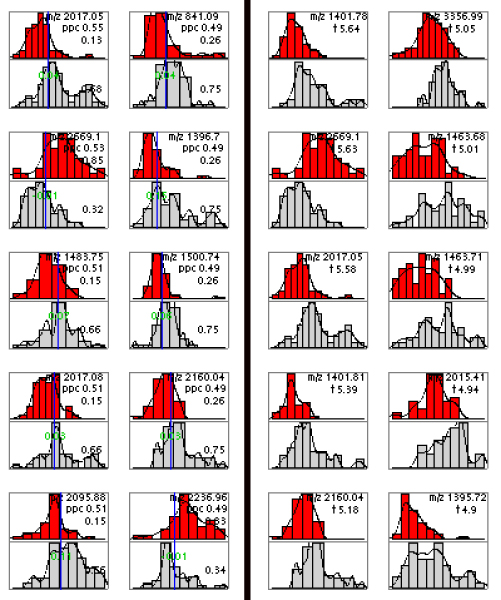
**Biomarker comparison**. Best 10 peaks (i.e. peptide signals) from the *Ovarian cancer *dataset selected using PPC (left) and t-statistics (right). Each small panel shows a histogram of peak heights in the training set at one *m*/*z *value for healthy patients (top) and cancer patients (bottom). Vertical line corresponds to the estimated optimal height split point (see [7] for details). The proportions of samples in each class having peaks higher than the split point are indicated (e.g. for top left corner panel these proportions are respectively 0.13 and 0.68). Notice that the two selected sets have only 3 peaks in common.

Appropriate feature selection for mass spectrometry data is crucial from diagnostic point of view. Selected peaks from MS datasets corresponding to peptide signals should be further studied in order to identify functional relationships and biological processes underlying the given disease. Peptides from proteins, which have clear biochemical functionality, can be treated as biomarkers for the disease. One typical approach to feature selection is to rank the features according to the value of some statistic. We examine four popular feature ranking criteria (t-statistic [10], mutual information [11], peak probability contrasts [7] and random forest variable importance measure [12]) and show that even though each of these methods is intuitively compelling, they result in different feature rankings (see Figure 1). Similar observations have been made in [7] and [8]. This stands in contradiction with the overall objective to use proteomic MS datasets for identification of biomarkers specific of a given disease. It is natural to postulate that one does not obtain many different biomarker lists for the same dataset. At the same time it is not obvious, which single criteria best describes a biomarker. We are inclined to assume that each scoring function provides unique information about the variables. Therefore, we propose to take into account information coming from various sources. This should also aid in eliminating false positive biomarkers, as they are less likely to be scored favorably by all of the distinct methods.

We present two novel solutions to the biomarker selection problem, both of which aim at unifying the preferences of a number of feature scoring functions. The first approach is based on computing a consensus ranking, given the individual rankings from several scoring functions. This is often referred to as rank aggregation. This problem was formulated in the context of the Web search engines in [13] and several heuristics have been proposed therein for this task. In our application the consensus is found as the stationary distribution of an appropriately defined Markov chain over the set of features.

A different way of reducing the dimension of the data is by feature extraction. Instead of selecting a subset of the original features, the aim is to construct a group of new features that optimize a given objective. Often the method of choice is principal component analysis (PCA), which retains as much of the original variance of the data as possible, with the condition that the output variables are uncorrelated. Typically PCA is applied to all of the variables to extract a small number of new variables, which convey the greatest amount of the variance. We argue that in the case of MS data (and many other similar applications) it is unrealistic to assume that the directions of the greatest variance of the data capture the differences between the observed health conditions. Instead many other factors, independent of the given patient classification, like age, diet, or sample processing may contribute to the variance of the data. For best results and interpretability we propose to apply PCA only to a selected group of features. This selection is based on the outcome of a number of scoring functions.

## Results and discussion

**Figure 2:**
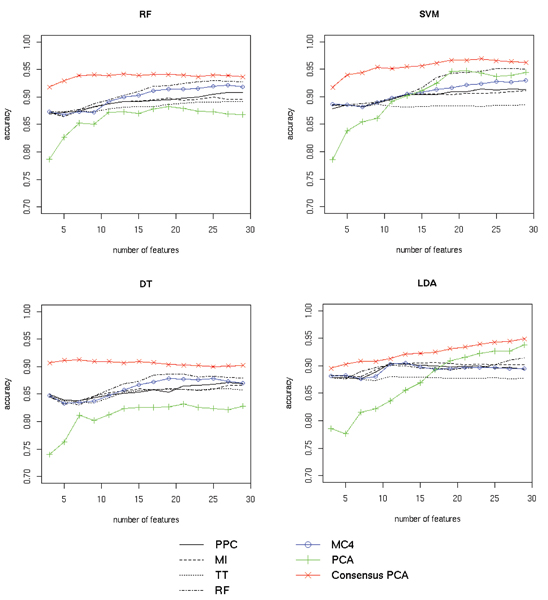
**Prostate cancer classification results**. Classification results for four classifiers (random forest (RF), SVM, decision trees (DT) and LDA) on the SELDI-TOF prostate cancer dataset are shown separately in the four panels. Classifier performance using a specified number of best features from individual scoring functions (peak probability contrast (PPC), mutual information (MI), t-statistic (TT) and random forest feature ranking (RF)) are plotted in black. Performance with features selected by MC_4 _rank aggregation of the four functions is shown in blue. Results for regular PCA and our modified "Consensus" version using only the best features from the four scoring functions are plotted in green and red respectively. For all methods the average accuracy (fraction of samples correctly classified) over 20 cross-validation runs is shown. See Section Results for discussion.

**Figure 3:**
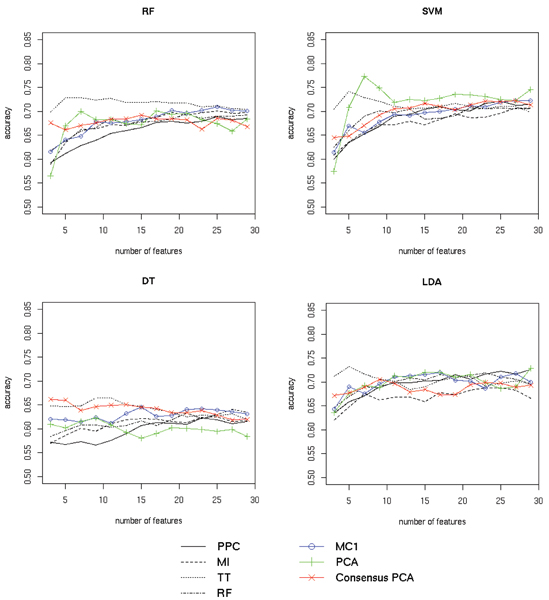
**Ovarian cancer classification results**. Classification results for four classifiers (random forest (RF), SVM, decision trees (DT) and LDA) on the MALDI-TOF ovarian cancer dataset are shown separately in the four panels. Classifier performance using a specified number of best features from individual scoring functions (peak probability contrast (PPC), mutual information (MI), t-statistic (TT) and random forest feature ranking (RF)) are plotted in black. Performance with features selected by MC_1 _rank aggregation of the four functions is shown in blue. Results for regular PCA and our modified "Consensus" version using only the best features from the four scoring functions are plotted in green and red respectively. For all methods the average accuracy (fraction of samples correctly classified) over 20 cross-validation runs is shown. See Section Results for discussion.

We have evaluated our methods on two MS datasets. The first dataset is a prostate cancer SELDI-TOF dataset provided by the Clinical Proteomics Program of the National Cancer Institute. This dataset contains 322 samples (63 samples from healthy donors, 190 from patients with benign prostatic hyperplasia (BPH), and 69 from patients with prostate cancer). The motivation was to be able to discriminate prostate cancer against other conditions, therefore we have decided to classify these samples into two groups: one with cancer samples and the other with all the rest. We note that overall better classification accuracy can be achieved on this dataset with the exclusion of BPH samples (data not shown). The second dataset used in our experiments is a MALDI-TOF ovarian cancer dataset from the Keck Laboratory at Yale University. We have obtained a preprocessed version of this dataset by contacting the authors of [8]. The dataset contains 91 samples (47 cancer and 44 control). Each sample contains intensity levels for 24262 peaks. All methods were evaluated by ten fold cross-validation in which all the steps (feature ranking, rank aggregation/PCA and supervised learning) were repeated for each fold on the training part of the samples. The average accuracy (fraction of samples correctly classified) is shown. Figures 2 and 3 show classification results for the prostate cancer and ovarian cancer datasets respectively.

### Markov chain rank aggregation

To evaluate the performance of the Markov chain rank aggregation algorithm we first scored each feature using the four scoring functions, thus obtaining four rankings. Next, we selected top 100 features from each list and aggregated the resulting partial rankings using the Markov chain method. Note that this process was repeated for each training block of the data in the cross-validation scheme. Best results were obtained using the MC_4 _transition matrix for the prostate cancer dataset and MC_1 _transition matrix in the case of the ovarian cancer dataset (the respective transition matrices are defined in Section Methods). Figures 2 and 3 compare the classifier accuracy (total number of correct class predictions divided by the total number of predictions) obtained using different feature selection methods and their aggregation for the two datasets. It can be observed that consensus ranking found by the MC rank aggregation performed well compared to the separate feature rankings. It did not achieve the highest overall score, but was clearly better than most of the input rankings it was computed from. In case of the prostate cancer dataset (see Fig. 2) the classification accuracy for features selected with MC_4 _closely followed the results for the best input method, while all other input methods performed much worse. In case of the ovarian cancer data (see Fig. 3), MC_1 _also outperformed three of four input methods. Best features selected with the t-statistic yielded the highest prediction accuracy on this dataset. However, the accuracy of most classifiers significantly decreased when more features from this ranking were used. The MC_1 _ranking displayed a more stable behavior increasing the prediction accuracy as more features were included.

### Consensus feature extraction *via *PCA

To evaluate the performance of the proposed consensus feature extraction method, for each cross-validation split we took a union of 100 best features from each ranking and applied PCA to the data reduced to only those features. A specified number of the outcome consensus features (sorted by the decreasing eigenvalues) was chosen each time to construct the classifier. Due to the limited number of the input variables, PCA outputs only a small number of significant features. We decided to take only those which constitute for at least 0.1% of the variance. The performance of standard PCA and the proposed "Consensus" version for the two datasets are presented in Figures 2 and 3. Features obtained from Consensus PCA for the prostate cancer dataset (see Fig. 2) were far more informative than the ones extracted using regular PCA (judging by classifier performance). The accuracy achieved using a small number of the consensus features also compared favorably with the results for a large set of features selected by the best input ranking function. In the case of the ovarian cancer data using Consensus PCA significantly improved the performance of the decision tree classifier. Remaining classifiers yielded better predictions using the standard PCA version. We also note that in the case of this dataset using standard principal components, which convey the variance of all features, overall resulted in more accurate predictions than using features selected by ranking methods (most evident with the SVM classifier). Based on these observations we suspect that in the case of this dataset lower ranked features provide additional information useful for class prediction. This would explain the lower accuracy of the classifiers constructed using the consensus features, which only convey information about the top 100 features from each method.

## Conclusion

We have proposed and tested two biomarker selection methods: one based on rank aggregation and second applying PCA to the informative variables selected by different scoring procedures. Both methods raise the possibility of identifying predictors supported by several competing feature ranking procedures. Although we focused on the analysis of MS data, our methods can just as well be applied to extract consensus predictors from other large-scale experiments (e.g. gene expression microarray data).

In order to confirm the relevance of predictors found by any computational means one must study their underlying biological function. We are currently involved in two proteomic projects carried out by the Laboratory of Mass Spectrometry of the Polish Academy of Science and the Warsaw Oncology Center, which aim at identifying prognostic biomarkers for cystic-fibrosis and diagnostic biomarkers for colorectal cancer. These studies will provide us a chance to validate our procedures with respect to their ability to identify more biologically meaningful predictors and less false positives.

## Methods

**Figure 4:**
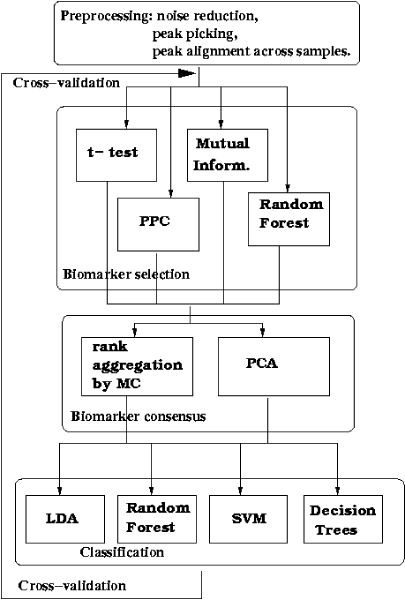
**Method overview**. The control flow through different phases of the proposed method: we start with a preprocessed MS dataset; apply several competing biomarker selection procedures (t-statistic, peak probability contrasts (PPC), mutual information and random forest feature ranking); achieve their consensus by the Markov chain rank aggregation method or PCA and train the classifiers (LDA, random forest, SVM, decision trees) on consensus features. For performance assessment the steps are repeated for each fold of the ten-fold cross-validation scheme.

In this section we describe the main steps of our approach (summarized in Figure 4). We focus our attention on feature selection and extraction for patient classification based on MS data. In order to carry out the presented procedures the raw MS require preprocessing, which usually includes background noise elimination, peak identification and cross-sample peak alignment. These steps will not be covered here (see e.g. [14-16] for methodological description and software for processing raw MS data). We will assume to work with an already preprocessed set of samples.

We will denote our dataset with a *p *by *n *matrix, where *p *is the number of intensity measurements and *n *is the number of samples. In a typical MS dataset the intensities correspond to *p *mass to charge (m/z) ratios, which determine the set of observed variables. Sometimes additional experimental techniques such as liquid chromatography (LC) are applied to distinguish between substances with the same mass, thus the observed variables may be further refined by these measurements. The rows of the data matrix contain the values of the observed variables (also called features) for each of the *n *samples. Given a set of training samples with known classification, our goal is to build a classifier which will be able to predict the class of a new sample. In the following discussion we will describe the subsequent steps leading to the construction of a classifier which uses consensus features (potentially biomarkers) computed by one of our methods. These steps include feature selection using various ranking functions, rank aggregation or consensus feature extraction and supervised learning.

### Biomarker selection by ranking functions

We begin by examining four feature selection methods. The first one considered here is the t-statistic (TT) [10], which measures the normalized differences of the means of a given feature in two groups (e.g. cancer and healthy group). The peak probability contrast method (PPC) for classification of MS samples was introduced in [7]. Here we exploit only the feature ranking part of the algorithm. It proceeds by determining for each feature the split point, which maximally discriminates between the two given groups of samples. The best split point is chosen among the quantiles of feature values (i.e. peak heights at a given position), (see [7] for details). As another way of determining feature importance, we consider the use of the mutual information measure (MI) [11]. Each peptide signal is treated as a discrete source which conveys information about the decision variable (class of the sample). Features with the greatest mutual information with the decision variable are considered the most important. The forth ranking statistic considered here is the feature importance measure computed by the random forest (RF) classification algorithm [12]. Note that we also use random forests as a classifier (see Section Classification).

**Figure 5:**
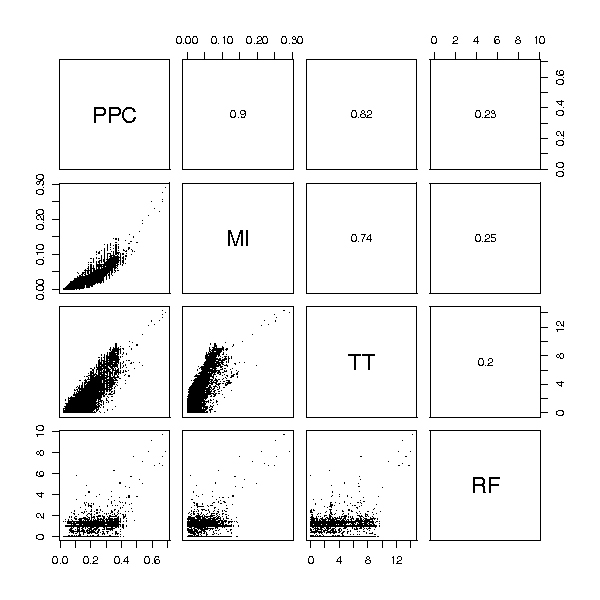
**Prostate cancer data ranking comparison**. Rankings obtained from several feature selection methods for the *Prostate cancer *dataset. Each small panel shows the comparison of two rankings. A point with coordinates (*i*, *j*) corresponds to the feature with score *i *in one method and score *j *in the other (for all methods the most important features receive the highest scores). Values of the Spearman correlation coefficient for each pair of scoring functions are given in the panels above the diagonal. The random forest feature ranking (RF) is considerably different than the rest. Peak probability contrast method (PPC), mutual information (MI) and t-statistic (TT) share a common group of the highest scored features, but significant differences can be observed in ranks of less important features, which also provide valuable information for classification.

**Figure 6:**
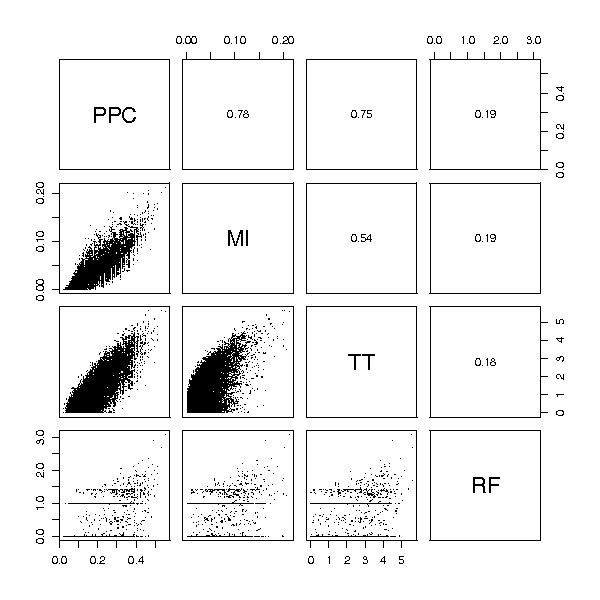
**Ovarian cancer data ranking comparison**. Comparison of rankings obtained from several feature selection methods for the *Ovarian cancer *dataset. Each small panel shows the comparison of two rankings. A point with coordinates (*i*, *j*) corresponds to the feature with score *i *in one method and score *j *in the other (for each method the most important features receive the highest scores). Values of the Spearman correlation coefficient for each pair of scoring functions are given in the panels above the diagonal. Notice significant differences between various rankings even within the group of the highest scored features. RF ranking stands out the most as in the case of prostate cancer data.

The typical approach to feature selection would be to use one of the above tests (or possibly a different method) to rank all the variables using the training samples and select top *k *of the variables (biomarkers) for further processing. One serious drawback of this approach is that different selection procedures result in different biomarker rankings (see Figures 5 and 6) and yield different classification rules. Each single selection method may provide valuable information about the features. Therefore it is reasonable to combine the information from several methods. In the following we describe two methods for aggregating the preferences of multiple scoring functions.

### Markov chain rank aggregation

The first approach proposed here is to compute an aggregated ranking from the outcomes of several feature selection procedures. The mathematical problem of rank aggregation was originally formulated in the context of Web search engines [13]. The idea is to start with several *partial rankings*, and produce one *consensus list *being the aggregation of them. Considered rankings are partial in two senses: several features, consecutive in the ranking list, can have the same label (i.e. they are equally ranked), and often we are interested only in the top *k *items from each list.

Rank aggregation problem has different formalizations based on various optimality criteria. Probably one of the most natural criteria – optimization of the average Kendall distance (the bubble sort distance between two lists) has been proven to be NP-hard in [13]. To cope with the complexity of the problem an efficient heuristic approach based on calculating the stationary distribution of an appropriately defined Markov chain has been proposed. The states of the chain correspond to the features ranked by various scoring functions and the transition probabilities depend on the position of the features in the given partial rankings. The aggregated consensus ranking is obtained as the list of states sorted by their stationary probabilities.

In this study we consider two Markov chains from [13] (MC_1 _and MC_4_). The transition matrices for the two Markov chains are defined as follows:

MC_1 _– If the current state is feature *P*, the next state is chosen uniformly from the multiset of all features that were ranked higher (or equal to) *P *by some feature selection method that selected *P*. The main idea is that in each step we move from the current feature to a better feature, allowing about 1k
 MathType@MTEF@5@5@+=feaafiart1ev1aaatCvAUfKttLearuWrP9MDH5MBPbIqV92AaeXatLxBI9gBaebbnrfifHhDYfgasaacH8akY=wiFfYdH8Gipec8Eeeu0xXdbba9frFj0=OqFfea0dXdd9vqai=hGuQ8kuc9pgc9s8qqaq=dirpe0xb9q8qiLsFr0=vr0=vr0dc8meaabaqaciaacaGaaeqabaqabeGadaaakeaadaWcaaqaaiabigdaXaqaaiabdUgaRbaaaaa@2F0B@ probability of staying in the same state, where *k *is roughly the average rank of the current feature.

MC_4 _– If the current state is *P*, then the next state is chosen as follows: first pick a state *Q *uniformly from the union of all ranked features. If *Q *is ranked better than *P *by the *majority *of the methods, that selected both *P *and *Q*, then go to *Q*, else stay in *P*.

In the case of chain MC_1_, we observed that its specific structure complies well with the framework developed in [17], where the Markov chain transition matrix *L *is assumed to have a form

**Figure 7:**
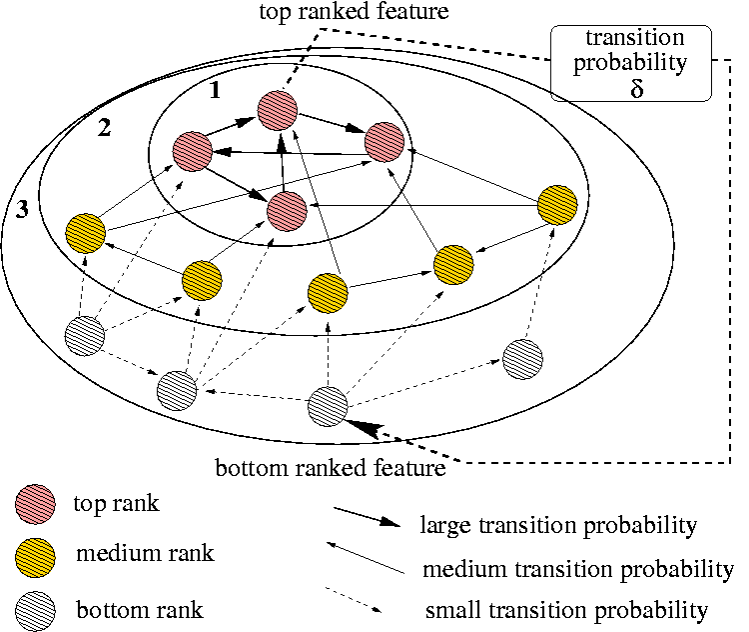
**Markov chain hierarchical structure**. The structure of the state space graph for rank aggregation Markov chain MC_1_. The type of edge corresponds to the transition probability. Ellipses surround the top ranked features appearing in each phase (from 1 up to 3 in this example). States joined at an earlier stage have higher stationary probability, and therefore rank higher in the aggregated ranking.

*L *= *L*_0 _+ *ε*·*L*_1 _+ *ε*^2^·*L*_2 _+ ... + *ε*^*k*^·*L*_*k*_,     (1)

for some parameter *ε *∈ (0, 1). This structure reflects the different strengths of interactions between the vertices of the Markov chain underlying graph (see Figure 7), and gives rise to an efficient algorithm for approximating the stationary distribution. The approximation algorithm is based on the idea of grouping together closely related states. Different interaction strengths (transition probabilities of the order of *ε*^*n*^, *n *= 1 ... *k *from Eq. (1)) result in several grouping phases. During the first grouping phase all the top ranked features (the ones that were never ranked lower than any other feature outside this group and have transition probabilities between themselves above a certain threshold) are lumped together. Second phase continues with the smaller state space and determines the next set of top ranked features, which are substituted by a single clump in the third phase, etc. States which are joined in the last phase have the lowest stationary probability. For each clump of grouped states the stationary distribution is calculated separately using the Grassmann-Taksar-Heyman (GTH) algorithm [18] and appropriately updated during the subsequent phases of the algorithm.

To ensure that all states will be grouped at some stage, we set the transition probability from the top to the bottom ranked features from each ranking list to a small value *δ *> 0. Recall that in the transition matrix of MC_1 _the probabilities from lower ranked features to higher ranked are also greater than 0. This ensures that the entire underlying graph is strongly connected, which is a prerequisite of the approximation algorithm. The probability value *δ *is chosen appropriately to guarantee that the corresponding interactions are taken into account during the last grouping phase, hence it has negligible influence on the final result.

The structure of the graph underlying the Markov chain MC_1 _is presented in Figure 7. Depicted is the nested family of sets grouped together during the consecutive phases of the algorithm.

Readers interested in the details of the approximation algorithm are referred to [19], (an extended version of it is available at [20]).

The hierarchical approach from [19] complies well with chain MC_1_. The structure of MC_4 _does not allow for efficient aggregation, but because of the limited number of features selected from each input ranking, it is still tractable. Table 1 summarizes the efficiency of Markov chain aggregation method. For MC_1 _we have 7 grouping stages; the sizes of resulting groups vary from 4 to 87. For MC_4 _all 242 states are grouped together in the first phase and the stationary distribution vector is calculated exactly using the GTH algorithm [18]. The significant speed-up obtained with the approximation algorithm is crucial when aggregation is applied to complete rankings of features from massive datasets.

### Consensus feature extraction

The second method proposed here is based on principal component analysis (PCA) [21]. PCA is a projection method, which seeks linear combinations of the original variables with maximal variance. The *i*-th projection vector (orthogonal to all previous projection vectors) is given by

vi=arg⁡max⁡α⊥v1,...,vi−1αTΣααTα,     (2)
 MathType@MTEF@5@5@+=feaafiart1ev1aaatCvAUfKttLearuWrP9MDH5MBPbIqV92AaeXatLxBI9gBaebbnrfifHhDYfgasaacH8akY=wiFfYdH8Gipec8Eeeu0xXdbba9frFj0=OqFfea0dXdd9vqai=hGuQ8kuc9pgc9s8qqaq=dirpe0xb9q8qiLsFr0=vr0=vr0dc8meaabaqaciaacaGaaeqabaqabeGadaaakeaaieqacqWF2bGDdaWgaaWcbaGaemyAaKgabeaakiabg2da9maaxababaGagiyyaeMaeiOCaiNaei4zaCMagiyBa0MaeiyyaeMaeiiEaGhaleaaiiGacqGFXoqycqGHLkIxcqWF2bGDdaWgaaadbaGaeGymaedabeaaliabcYcaSiabc6caUiabc6caUiabc6caUiabcYcaSiab=zha2naaBaaameaacqWGPbqAcqGHsislcqaIXaqmaeqaaaWcbeaakmaalaaabaGae4xSde2aaWbaaSqabeaacqWGubavaaacceGccqqFJoWucqGFXoqyaeaacqGFXoqydaahaaWcbeqaaiabdsfaubaakiab+f7aHbaacqGGSaalcaWLjaGaaCzcamaabmaabaGaeGOmaidacaGLOaGaayzkaaaaaa@57DB@

where **Σ **is the covariance matrix. The solution to (2) is given by the eigenvector of **Σ**, which corresponds to the *i*-th largest eigenvalue. PCA is particularly useful for reduction and interpretation of biological data from high-throughput technologies like mass spectrometry and micro-arrays. It is an unsupervised method, so it does not take into account the classification of the samples. As a characteristic of high-throughput biological experiments, large part of the variance of the data is possibly unrelated to class assignments. Various biological and processing factors can contribute to the overall diversity of the samples. Hence, for classification purposes, the aim should not be to preserve the overall variance, but rather to preserve the variance between classes. In order to boost the discrimination power of the extracted features, we apply PCA only to the group of the most discriminative variables. This also allows for easier interpretability of the resulting features (i.e. determining the contributions of the original variables), as the linear combinations are taken over a much smaller set of vectors. We take the union of the sets of top *k *features from each of the considered feature selection methods to generate the set of discriminative features and apply PCA to the reduced data matrix to extract a set of uncorrelated *consensus features*.

### Classification

We use four well-known supervised learning algorithms in our experiments, namely: linear discriminant analysis (LDA), support vector machines (SVM), random forests (RF), and decision trees (DT). All these methods have been compared in [8]. We provide only a brief description of SVM and RF below. For details and description of LDA and DT see e.g. [22,23].

#### Support Vector Machines

Support Vector Machines were introduced by Vapnik [24]. The method seeks the optimal hyperplane separating two classes. In the case of linearly separable data, the optimal hyperplane can be found by solving a linear optimization problem. Often the training points are not perfectly linearly separable. SVM deals with this problem by mapping the data points into higher-dimensional space. For details refer to [25].

#### Random forest

It has been found that aggregating classifiers built from perturbed versions of the training set could substantially improve prediction accuracy. Random forest ([12]) is a very effective classifier which exploits this idea, by constructing an ensemble of classification trees and basing the decision on the majority vote. Each tree is build on a bootstrap sample of the training data and random feature selection is applied at each node of the tree.

## Abbreviations

• **BPH **– Benign Prostatic Hyperplasia

• **DT **– Decision Trees

• **GTH **– Grassmann-Taksar-Heyman Algorithm

• **LC **– Liquid Chromatography

• **LDA **– Linear Discriminant Analysis

• **MALDI-TOF **– Matrix-Assisted Laser Desorption/Ionization Time-of-Flight Spectrometry

• **MC **– Markov Chain

• **MI **– Mutual Information

• **MS **– Mass Spectrometry

• **PCA **– Principal Component Analysis

• **PPC **– Peak Probability Contrasts

• **RF **– Random Forest

• **SELDI-TOF **– Surface-Enhanced Laser Desorption/Ionization Time-of-Flight Spectrometry

• **SVM **– Support Vector Machines

• **TT **– T-Test

## Competing interests

The authors declare that they have no competing interests.

## Authors' contributions

Both authors participated in the design of the approach, contributed to the manuscript, read and approved its final version. JD performed the biomarker selection and classification experiments and AG provided the implementation of the hierarchical algorithm for approximating MC stationary distribution.

## Figures and Tables

**Table 1:** Markov chain grouping comparison.

MC	# stages	groups sizes	time (in sec)
MC_1_	7	4, 15, 46, 51, 87, 14, 25	1.8
MC_4_	1	242	21.7

Performance of the hierarchical aggregation algorithm on two versions of the transition matrix (MC_1 _and MC_4_) for the *Ovarian cancer *dataset. For both considered Markov chains the number of phases, group sizes, and running times are given. In the case of MC_1 _the algorithm exploits the hierarchical structure of the chain to speed up the stationary distribution calculation.
